# Long-term outcomes of mitral valve replacement in patients weighing less than 10 kg

**DOI:** 10.1186/s13019-021-01443-9

**Published:** 2021-03-31

**Authors:** Haoyong Yuan, Zhongshi Wu, Ting Lu, Yilun Tang, Jinlan Chen, Yifeng Yang, Can Huang

**Affiliations:** 1 Department of Cardiovascular Surgery, The Second Xiangya Hospital of Central South University, Changsha, 410011 Hunan China; 2 Engineering Laboratory of Hunan Province for Cardiovascular Biomaterials, 139# Renmin Road, Changsha, Hunan 410011 P.R. China

**Keywords:** Mitral valve replacement, Pediatric, Mechanical prosthesis, Lower cardiac output syndrome

## Abstract

**Background:**

The outcomes of mitral valve replacement (MVR) in pediatrics especially in the patients weighing less than 10 kg are not always favorable. This study aimed to measure long-term outcomes of MVR in our institution.

**Methods:**

Nine young children weighing less than 10 kg underwent MVR with mechanical prostheses were enrolled in this retrospectively study. Kaplan–Meier survival analysis was used for the prediction of freedom from death and adverse events. Chi-square test was performed to compare outcomes for patients with different ratios of mechanical prosthesis size and body weight. Fourteen related literatures were also reviewed to support our study.

**Results:**

All patients received bileaflet mechanical prostheses replacement. The surgical technique varied among the patients with prostheses implanted in the intra-annular (*n* = 5), supra-annular (*n* = 1), or with a Dacron conduit segment in the supra-annular position (*n* = 3). The valve size/weight ratio ranged from 2.11 to 5.00. There were two early death and one late death post-operation. The mean follow-up period was 80.67 ± 63.37 months, the transvalvular gradient was 10.5 ± 1.76 mmHg (range 8 to 12) and the peak gradient of LVOT was 5.00 ± 0.64 mmHg. One (11.1%) patient underwent an immediate revision MVR after initial MVR due to the periprosthetic leak. No patients required surgical reintervention or permanent pacemaker placement during long-term follow-up.

**Conclusions:**

The tailored surgical strategy utilized for MVR in infants resulted in reliable valve function and excellent survival. Although revision is inevitable due to somatic growth, the bileaflet mechanical prostheses displayed appropriate durability.

**Supplementary Information:**

The online version contains supplementary material available at 10.1186/s13019-021-01443-9.

## Visual abstract

- Key question

What should we do for patients with mitral valve diseases weighing less than 10 kg?

- Key findings

Mechanical valve prostheses might be a good choice.

- Take-home message

MVR in infants resulted in reliable valve function and excellent survival. The mechanical prostheses displayed appropriate durability.

## Introduction

Mitral valve diseases in children are complex and are always accompanied by various cardiac anomalies, making surgical treatment challenging [1]. Although the techniques of mitral valve repair (MVP) in children have been well established and provide acceptable long-term outcomes, mitral valve replacement (MVR) remains the sole surgical alternative [2]. Because of early calcification and short durability of bio-prostheses, mechanical mitral valve replacement (mMVR) is the most common choice for the pediatric population, especially for neonates and infants [3]. In these cases, a larger-sized prosthesis valve is implanted into the small annulus, possibly causing high mortality, left ventricular outflow obstruction (LVOTO), complete atrioventricular block postoperatively, pulmonary vein stenosis, low valve durability and need for anticoagulant management postoperatively [4]. During the past decades, many techniques and prosthesis have been employed to treat mitral valve diseases in infants and the outcomes of the MVR in patients weighing less than 10 kg are not always favourable. To better understand the optimal approach to this complex problem, we retrospectively examined our experience with children less than 3 years old and weighing less than 10 kg who underwent mMVR.

## Patients and methods

### Patients

We considered all patients weighing 10 kg or less and younger than 3 years old who underwent MVR with mechanical prostheses at the Second Xiangya Hospital of Central South University in China between November 2006 and April 2019. Data were acquired from hospital records and the institute’s cardiac database and were analyzed retrospectively. This study was approved by the Institutional Ethics Committee and written informed consent was obtained from parents or guardians before surgery to allow the use of their data.

### Surgical technique

**Fig. 1:**
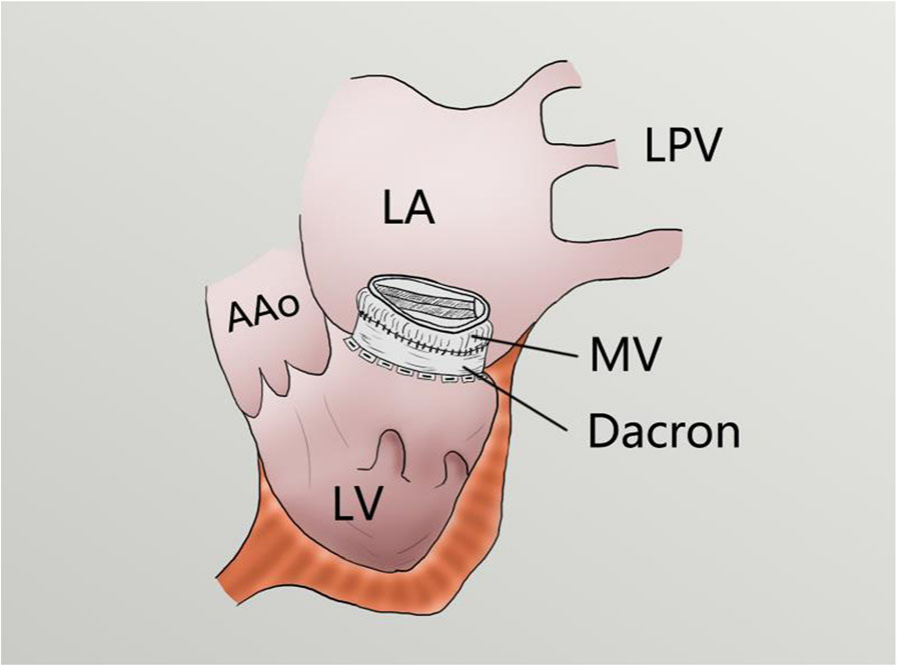
Valves implanted supra-annularly with a tilt

All patients underwent median sternotomy and standard cardiopulmonary bypass under moderate hypothermia (range 28 to 32 °C). The mitral valve was exposed through an “L” shaped atrial septectomy (from the entrance of superior vena cava to the front of the coronary sinus). mMVR was performed after unsuccessful mitral valvuloplasty or when the valve was judged to be unsuitable for repair. The posterior leaflet tissue and subvalvular apparatus of the mitral valve were preserved wherever possible. In patients with infective endocarditis, all valve tissue was excised and sent for bacterial cultures. Valves were implanted in either a supra- or intra-annular position; supra-annular valves were sometimes tilted either anteriorly or posteriorly to optimize positioning. When doing the skirt super-annular MVR, a segment of Dacron conduit was interruptedly sutured in the annular position and then the mechanical prosthesis was running sutured to the conduit (Fig. 1 and Video). Three patients underwent MVR concomitant with the repair of the associated cardiac abnormalities, including ventricular septal defect (VSD) closure (*n* = 1), ligation of patent ductus arteriosus (PDA) (*n* = 1), correction of coarctation of the aorta (*n* = 1). Intraoperative transesophageal echocardiography was used to confirm adequate prosthetic valve function in all cases.

### Follow-up

Postoperatively, anticoagulation was obtained using sodium warfarin, aiming at an international normalized ratio (INR) of 2.0–2.5. Temporary anticoagulation with intravenous heparin was also used during the 24 h after the surgery and invasive procedures, aiming at an activated partial thromboplastin time (APTT) of 1.5–2.0 times above baseline.

### Statistical analysis

The Statistical Package for Social Sciences (SPSS) version 22.0 (IBM) was used for all data analyses. Descriptive statistical analysis was undertaken using continuous data presented as median and interquartile range (IQR) or mean ± standard deviation and categorical variables as raw data and/or percentages. Kaplan–Meier survival analysis was used for the prediction of freedom from death and adverse events. Chi-square test was performed to compare outcomes for patients based on the ratio of mechanical prosthesis and weight.

## Result

### Demographics

A total of nine pediatric patients were included in this study; there were three boys and six girls in this cohort. The ages of seven patients were less than 1 year at the initial mMVR procedure. The mean age at the initial mMVR was 11.88 ± 11.29 months (range 1 months to 32 months) and the mean body weight was 6.83 ± 2.56 kg (range 4.0 to 9.5 kg). Two patients weighing less than 5 kg were diagnosed with severe congenital mitral valve regurgitation and stenosis and were treated with ventilator before the operation. Three patients were diagnosed with severe mitral valve regurgitation with coarctation (CoA), patent ductus arteriosus, or ventricular septal defect (Table 1). Four (44.4%) patients underwent previous surgical procedures, of which three underwent partial or complete AVSD repair and one underwent VSD closure and MVP, one of which was accompanied by fungal endocarditis after the partial AVSD repair.

**Table 1:** Patients list

NO.	Sex	Age	Weight	Diagnosis	Procedure	VALVE (SIZE)	Ratio	Redo	Clamping	Bypass	Ventilation time	ICU Stay	PG 1 week post	Death
1	F	9	9	MR, Post CAVSD Repair	SSMVR	19	2.11	N	173	285	66	3.3	8	N
2	M	3	5.5	MR, VSD	SMVR	19	3.45	Y	125	185	336	14		Y
3	F	3	4.7	MS, MR	SSMVR	19	4.0	N	104	121	124	10	12	N
4	F	1	3.8	Post MVP, MR	MVR	19	5	N	160	209	48	2		Y
5	F	12	7	IE, MR, Post PAVSD Repair	MVR	21	3	N	56	95	504	89		Y
6	F	21	9	MR, PDA	MVR	25	2.7	N	33	57	19	1.8	12	N
7	M	2	4	MS, MR	SSMVR	16	4	N	124	171	384	20	12	N
8	F	32	9.5	MR, Post PAVSD Repair	MVR	25	2.6	N	104	155	72	7	10	N
9	M	24	9	MR, CoA	MVR	25	2.7	N	56	111	120	8	9	N

Age (months), Clamping and Bypass (minute), Ventilation time (hour), ICU Stay (day), Weight (kg), Size (mm), PG (mmHg), *F* Female, *M* Male, *N* NO, *Y* Yes, *Ratio* Valve size/weight, *Redo* Reintervention after initial MVR, *MR* Mitral valve regurgition, *MS* Mitral valve stenosis, *VSD* Ventricular septal defect, *PDA* Patent duct arteriol, *IE* Infective endocardiatis, *PAVSD* Partial artroventricular septal defect, *CAVSD* Complete artroventricular septal defect, *CoA* Coarctation, *MVP* Mitral valve repairment, *MVR* Mitral valve replacement, *SMVR* Supra-annul mitral valve replacement, *SSMVR* Skirt supra-annul mitral valve replacement, *PG* Pressure gradient

### Surgical technique

**Fig. 2:**
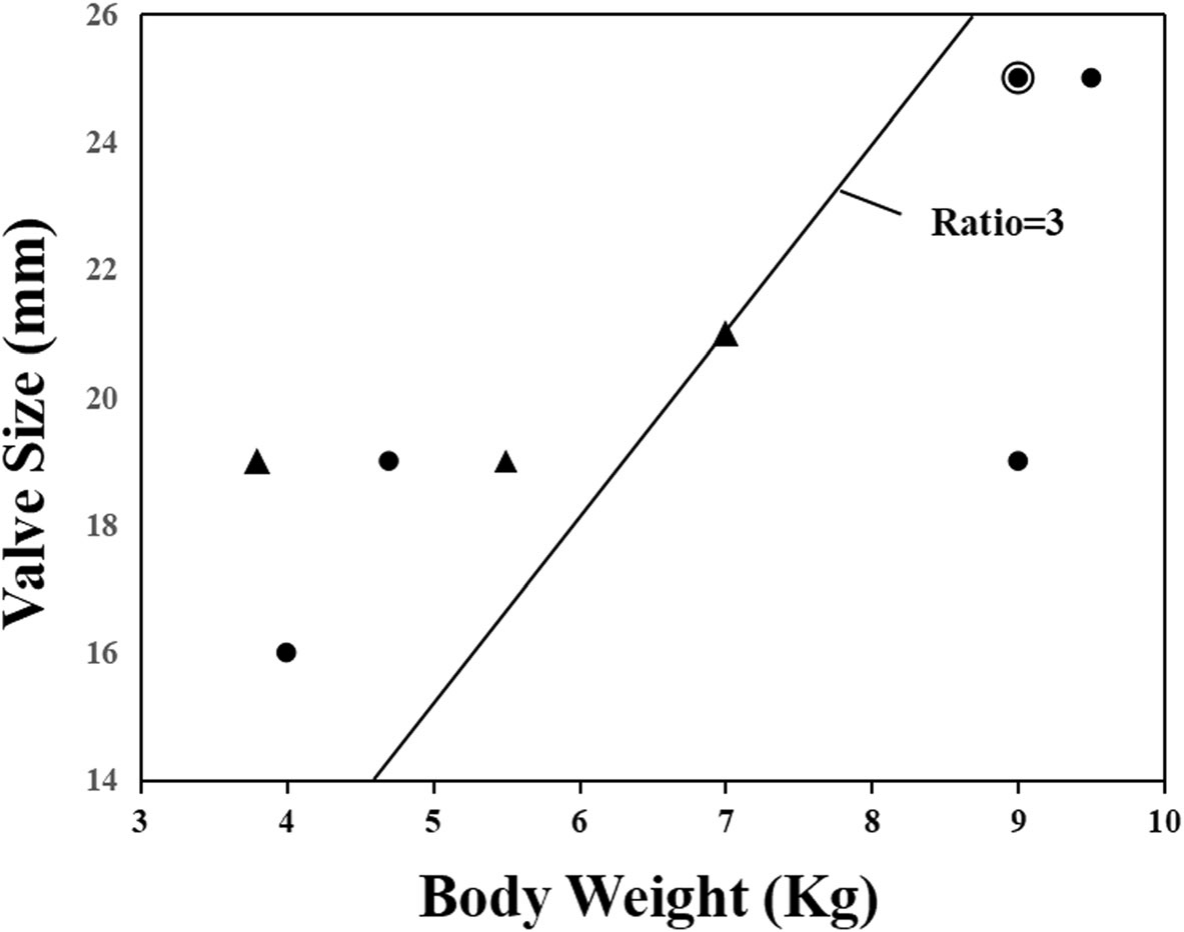
The patients Ratio between valve size/body weight

All patients received bileaflet mechanical prosthetic valves (ATS Medtronic, Minneapolis, Minnesota), with three receiving mitral prostheses and six receiving inverted aortic prostheses. Surgical technique varied between patients with valves implanted intra-annularly (*n* = 5), supra-annularly (*n* = 1), or supra-annularly with a segment of Dacron conduit (*n* = 3). The valve size/weight ratio ranged from 2.11 to 5.00 (average 3.28 ± 0.91) and there is significant difference between the ratio over 3 and below (*p* = 0.03) for early mortality (Fig. 2 and Table 2). The cardiopulmonary bypass time was 154.33 ± 68.25 min (range 57 min to 285 min), and the aorta clamp time was 103.89 ± 47.94 min (range 33 min to 173 min). At our institution, patient INRs were strictly monitored at outpatient hematology clinics during follow-up; there were no emergency operations for acute valve thrombosis or a significant event in other series.

**Table 2:** Comparison of the survival between ratio ≥ 3 and below

	Death	Survival	*P value*
**Ratio ≥ 3**	3	2	*P* = 0.03
Ratio < 3	0	4	

### Early and long-term follow-up outcomes

**Fig. 3:**
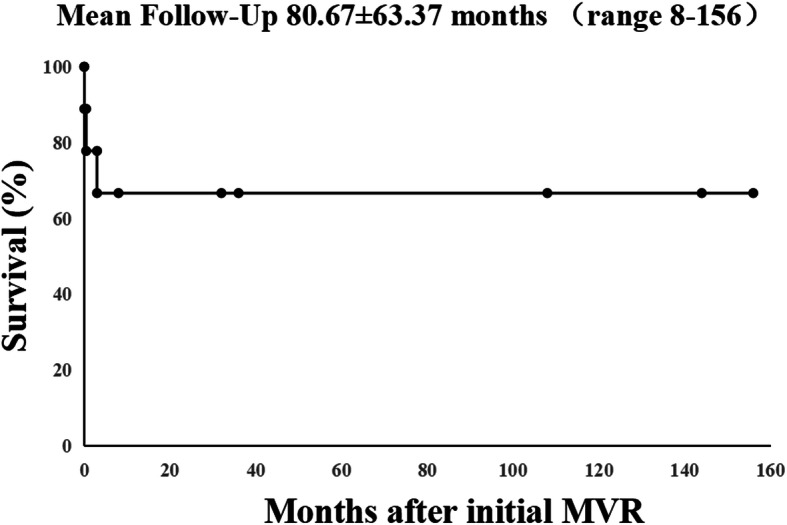
Kaplan-Meier analysis of survival

There were two early death within 1 month and one late death post-operation, all the three dead cases received intra-annular or supra-annular MVR. One died from hematencephalon which was secondary to fungal endocarditis and the other two died from low cardiac output syndrome (Fig. 3). One underwent redo MVR during hospital stay because of a perivalvular leak. The postoperative intensive care unit (ICU) stay ranged from 1.8 to 89 days with a median of 8 days. The median duration of postoperative ventilation was 120 h ranged from 19 to 504 h (Table 3). After a mean follow-up of 80.67 ± 63.37 months, the transvalvular gradient was 10.5 ± 1.76 mmHg (range 8 to 12) and the peak gradient of LVOT was 5.00 ± 0.64 mmHg. No patients required surgical reintervention for the development of left ventricular outflow tract obstruction or permanent pacemaker placement during long-term follow-up (Table 4).

**Table 3:** Patients character list

Age at operation	11.88 ± 11.29 months (range 1 months to 32 months)
Weight at operation	6.83 ± 2.56 kg (range 4.0 to 9.5 kg)
Interval from original ration	80.67 ± 63.37 months
The cardiopulmonary bypass time	154.33 ± 68.25 min (range 57 min to 285 min)
The aorta clamp time	103.89 ± 47.94 min (range 33 min to 173 min)
Duration of ventilation	Median 120 h (ranged from 19 to 504 h)
Duration of ICU stay	Median 8 days (ranged from 1.8 to 89 days)

**Table 4:** Follow up for the latest echocardiograft examination

Patients	Follow Up	PG (LVOT)	PG (MV)	mPAP
1	8	5	8	20
3	32	5	11	16
6	108	6	12	20
7	36	5	12	18
8	144	5	11	17
9	156	4	10	16

Follow up (months), *MV* Mitral valve, *PG* Pressure gradient, *LVOT* left ventricular outflow tract, *mPAP* Mean pulmonary artery pressure

## Discussion

MVP is considered to be the current standard treatment for children with mitral valve abnormalities. Unfortunately, there are certain pediatric patients who will need an MVR instead of an unsuccessful MVP; difficulties for both decision-making and treatment options may arise [5]. The most common indications for MVR in children include rheumatic disease, endocarditis, mitral stenosis in Shone’s syndrome or failed atrioventricular septal defect (AVSD) repair. In our studies, over 50% of the patients were diagnosed with mitral valve disease with infective endocarditis or failed AVSD repair. Bioprostheses were not the appropriate choices for MVR in children and infants due to the lack of durability and unavailability of small-sized prostheses. The pulmonary autografts and Contegra conduits were employed to treat pediatric patients with small annular to avoid long-term anticoagulation. However, because of accelerated degeneration and calcification, these techniques require long-term follow-up [6, 7]. Similar to the Ross procedure, the Ross II procedure makes one-valve problem to two-valve problems, possibly resulting in early regurgitation due to the lack of valve commissural support and higher trans-valvar pressure gradiant. Considering the better durability, availability, and hemodynamic performance, mechanical valves are the preferred mitral valve substitute in children.

Historically, MVR in infants has been associated with significant morbidity and mortality and long-term survival is lower than that of infant MV repair [8]. Consequently, surgical techniques and strategies have evolved to optimize outcomes. The reported operative mortality for MVR in infants is 5 to 30% and the 10- and 30-year survival for these patients was recently reported up to 75% [5] (Table 5). Heart block requiring pacemaker implantation, endocarditis, thrombosis, stroke, an increased ratio of prosthetic size/weight and supra-annular position were all found to be statistically significant predictors of early mortality [9]. In our institute, there were two early death within 1 month and one late death. Among the three dead cases, one died from hematencephalon secondary to fungal infection and two died from low cardiac output syndrome. The last two patients received relatively large prosthesis, and the size/body weight ratio was 3.45 and 5, respectively, which accord closely with the results reported by Caldarone et al. [1]. Although there were a relatively small number of cases in our study, we found that smaller annulus, heart failure before the procedure and fungal infections were risk factors for short-term mortality. Previous studies demonstrated that age less than 2 years old at MVR was a risk factor for operative mortality [20]. Rafii et al. found that there was no significant difference in survival between patients aged less than 2 years and patients aged 2 to 18 years, and age less than 2 years remained a risk factor for reoperation but not for mortality [13]. Bileaflet mechanical prostheses from ATS Medtronic (Minneapolis, Minnesota) were implanted in nine patients in our study. Because the smallest size of the available mechanical mitral valve in our institute is 25 mm, six patients were implanted with mechanical aortic valve prostheses. Due to the low profile, excellent hemodynamics and good durability, a bileaflet mechanical valve is the prosthesis of choice in the mitral position in children [18]. Size mismatch between the mechanical prosthesis and mitral valve annulus is considered to be a risk factor for operative mortality [21]. Caldarone et al. showed that 1-year survival rate was 91% when the prosthesis size–to–patient body weight ratio was < 2; however, the survival rate was only 61% when the ratio > 4 and only 37% when the ratio < 5 [1]. In our study, the ratio ranged from 2.1 to 5 and the ratios of the deceased patients were all over 3. This suggests that an appropriate mechanical prosthesis is essential for successful MVR in children. Prosthesis size should be carefully chosen based on the body weight, age, and mitral valve annular size of an individual patient.

**Table 5:** Literature review of long-term survival and freedom from redo MVR after MVR

Studies	Cases	Age	Follow up	Survival rate	Free from Redo MVR
Mater, Kathryn. 2019. Australia [9]	22	Mean age 6.8 ± 4.1 months	6.2 ± 4.4 years	100%	86.1% at 1 years, 80.7% at 5 years and 21.2% at 10 years
Raffaele Giordano. 2015. Italy [2]	7	Mean age 13.3 ± 11.2 months	67.1 ± 34.8 months	100%	71.4%
Christopher A. Caldarone .2015. USA [1]	139	Mean age 1.9 ± 1.4 years	Median 6.2 years	74%	
Jiyong Moon. 2015.J apan [10]	18	Mean age 4.0 ± 1.8 months	4.5 ± 3.8 years	89.1%	57.8% at 10 years
John W. Brown. 2012. USA [11]	97	Median age 8 years	12.8 ± 10.1 years	71%	94% at 1 year, 82% at 5 years, 71% at 10 years, and 63% at 20 and at 35 years
Hyung-Tae Sim. 2012. Korea [12]	19	Mean age 7.6 ± 5.5 years	76 ± 56 months	100%	94.7 ± 5% at 10 years
Daniela Y. Rafifii. 2011. USA [13]	18	Median age 1.2 years	Median 5.4 years	82%	69% at 5 years and 40% at 10 years
Kirk R. Kanter. 2011. USA [14]	15	Mean age 337 ± 412 days	4.3 ± 2.8 years,	84%	69% at 5 years and 21% at 10 years
Bahaaldin Alsoufi. 2009. Canada [15]	79	Median age 24 months	4.1 ± 3.7 years	62%	
ElifSeda Selamet Tierney. 2008. USA [8]	118	Median age 16.3 months	Over 30 years	56%	72% at 5 years and 45% at 10 years
J. S. Sachweh. 2007. Germany [16]	17	Mean age 4.3 ± 4.3 years	9.1 ± 6.6 years	94.1%	93.4% at 1 year 89.0% at 5 and 10 years
Wolfram Beierlein. 2007. UK [17]	54	Median age 3.0 years	Median 9.2 years	33%	45.3% at 5 years and 17.3% at 10 years
Hunaid A. Vohra. 2007. UK [18]	24	Mean age 1.4 ± 1.3 years	Median 7.5 years	75.7%	
Naoki Wada. 2005. Japan [19]	18	Mean age 1.02 ± 0.72 years	3.3 ± 3.5 years	68.9%	87.1% at 5 years and 69.6% at 10-years

Multiple surgical techniques were employed in the MVR. The appropriate mechanical valve was implanted in the annulus if the size matched. Because of the link between the mechanical valve size and freedom from redo MVR, a large mechanical prosthesis was implanted to the smaller annulus, possibly causing atrioventricular block and left ventricle outflow tract obstruction related to valve impingement on surrounding cardiac structures [22]. In the neonate or infant with a small native annulus, implantation of commercially available prosthetic valves in the annular position can be problematic. Placing the prosthesis in a supra-annular position is an alternative when a more traditional annular implantation is not possible. The prosthetic valve was implanted with interrupted pledget polyester sutures with the pledgets on the atrial side of the prosthesis [10]. Previous publications suggested that the early results with supra-annular MVR in children were discouraging and identified it as a risk factor for early mortality because of the reduction of LA volume and compliance and aneurysm formation in the segment of LA between the prosthesis and the annulus [2]. One of our patients had valves implanted with a tilt, similar to that described by Moon and colleagues [10], which involved suturing part of the valve onto the native annulus and the remainder to the left atrial wall or atrial septum. The prosthesis was thereby implanted supra-annularly with a tilt either anteriorly or posteriorly to prevent impingement on the LVOT, pulmonary vein orifices, and conduction tissue. Two weeks later, the patient underwent redo MVR because of the periprosthetic leakage and died from acute low cardiac output syndrome. We suggest that implanting the prothesis supra-annularly with a tilt may have caused the periprosthetic leakage and the immediate redo MVR. Three patients had Dacron Hemashield (Meadox Medicals, Inc., Oakland, NJ) with interrupted sutures sewn to the native valve annulus, after which the prosthetic valve was sewn with running sutures into the conduit, among which two had a size/body weight ratio over 4. We employed the Dacron conduit that would be softer and provide better hemodynamics than the Gore-Tex conduit. The prosthetic valves were implanted to the conduit follow suturing the conduit wall to the annulus, which may provide convenience for the surgeon to implant a larger valve in a smaller space, also reducing the occurrence of periprosthetic leakage [23]. The avoidance of directly suturing on mitral valve annulus or left atrial wall may contribute to eliminating excessive traction of the left atrial tissue, left circumflex coronary artery, and pulmonary vein orifices. This surgical technique might be more tolerant to large prosthesis size according to our experience. The technique of intermittent suture would preserve the growth potential and may provide the possibility for the replacement of a larger mitral valve in the future. The mitral valve on the annulus will inevitably lead to the reduction of the left atrium content, possibly leading to pulmonary vein obstruction or even pulmonary hypertension. However, in our case, we did not find the existence of pulmonary vein stenosis or pulmonary hypertension. In fact, either mitral regugitation or mitral stenosis patients has a dilated left atrium. We believe that the larger left atrium has sufficient space for buffering and grasping the height of the Dacron conduit that can effectively avoid this complication.

Redo valve replacement is inevitable following infant MVR because of somatic growth. The duration has been reported to be 8.6 ± 6.6 years in children < 5 years of age at initial MVR and 7.3 years following infant MVR [24]. The most common reported indication for early redo valve replacement is excessive pannus formation, particularly in infants and young children. Valve type, size, and positioning were thought to optimize the longevity of the implanted prosthesis and maximize time until redo MVR [11]. As presented in our follow-up results, the transvalvular gradients of the implanted mechanical aortic valve and mitral valve demonstrated no significant differences. Studies indicated that choosing a mechanical valve larger than 19 mm could considerably delay the redo MVR [5], due to valve size ≥19 mm. There were no redo MVRs for somatic growth in our cohort.

There are several limitations to this study. First, it was a single-centre study, and therefore may be subject to selection bias. For this reason, we instituted strict inclusion and exclusion criteria. Multi-centre studies are needed to validate our findings. Bedsides that, study is limited by its retrospective design and the relatively small patient population.

## Conclusion

In terms of long-term outcomes, MVR is a good alternative for failed MVP in infants and young children whose weight is less than 10 kg. The ratio between the size of mechanical prosthesis and weight is a risk factor for patients undergoing mechanical MVR. The size of mechanical vale over 19 mm can avoid redoing MVR for 10 years or more. The skirt super-annular may reduce the mortality associated with MVR with a mismatched ratio.

## Supplementary Information

**Additional file 1: Video**.

## Data Availability

The data sets supporting the results of this article are included within the article and its additional files.

## Abbreviations

MVP: Mitral Valve Repair
MVR: Mitral Valve Replacement
mMVR: Mechanical Mitral Valve Replacement
SMVR: Supra-Annul Mitral Valve Replacement
SSMVR: Skirt Supra-Annul Mitral Valve Replacement
LVOTO: Left Ventricular Outflow Obstruction
INR: International Normalized Ratio
APTT: Activated Partial Thromboplastin Time
SPSS: Statistical Package for Social Sciences
IQR: Interquartile Range
ICU: Intensive Care Unit
AVSD: Atrioventricular septal defect
MR: Mitral Valve Regurgition
MS: Mitral Valve Stenosis
VSD: Ventricular Septal Defect
PDA: Patent Duct Arteriol
IE: Infective Endocardiatis
PAVSD: Partial Artroventricular Septal Defect
CAVSD: Complete Artroventricular Septal Defect
CoA: Coarctation
PG: Pressure Gradient
mPAP: Mean Pulmonary Artery Pressure
